# Habenula as a Possible Target for Treatment-Resistant Depression Phenotype in Wistar Kyoto Rats

**DOI:** 10.1007/s12035-022-03103-y

**Published:** 2022-11-08

**Authors:** Agata Korlatowicz, Paulina Pabian, Joanna Solich, Magdalena Kolasa, Katarzyna Latocha, Marta Dziedzicka-Wasylewska, Agata Faron-Górecka

**Affiliations:** Department of Pharmacology, Maj Institute of Pharmacology, Polish Academy of Sciences, Smętna 12, 31-343 Kraków, Poland

**Keywords:** Treatment-resistant depression, Habenula, miRNA, mRNA, Serotonin receptor 5-HT7, Potassium chloride channel KCC2, Wistar Kyoto rats

## Abstract

The mechanisms of treatment-resistant depression (TRD) are not clear and are difficult to study. An animal model resembling human TRD is the Wistar Kyoto rat strain. In the present study, we focused on selecting miRNAs that differentiate rats of the WKY strain from Wistar Han (WIS) rats in two divisions of the habenula, the lateral and medial (LHb and MHb, respectively). Based on our preliminary study and literature survey, we identified 32 miRNAs that could be potentially regulated in the habenula. Six miRNAs significantly differentiated WKY rats from WIS rats within the MHb, and three significantly differentiated WKY from WIS rats within the LHb. Then, we selected relevant transcripts regulated by those miRNAs, and their expression in the habenular nuclei was investigated. For mRNAs that differentiated WKY rats from WIS rats in the MHb (*Cdkn1c*, *Htr7*, *Kcnj9*, and *Slc12a5*), their lower expression correlated with a higher level of relevant miRNAs. In the LHb, eight mRNAs significantly differentiated WKY from WIS rats (upregulated *Htr4*, *Drd2*, *Kcnj5*, and *Sstr4* and downregulated *Htr2a*, *Htr7*, *Elk4*, and *Slc12a5*). These data indicate that several important miRNAs are expressed in the habenula, which differentiates WKY rats from WIS rats and in turn correlates with alterations in the expression of target transcripts. Of particular note are two genes whose expression is altered in WKY rats in both LHb and MHb: *Slc12a5* and *Htr7*. Regulation of KCC2 via the 5-HT7 receptor may be a potential target for the treatment of TRD.

**Supplementary Information:**

The online version contains supplementary material available at 10.1007/s12035-022-03103-y.

## Introduction

Major depressive disorder (MDD) is a highly prevalent and debilitating disorder that continues to increase the number of mentally ill individuals independent of age and sex. MDD symptoms are characterized by depressed mood; diminished interests; loss of experiencing pleasure; impaired cognitive function; and other symptoms, such as disturbed sleep or appetite [1]. Treatment of MDD patients includes psychotherapy, psychopharmacology, or a combination of the two approaches. Many different antidepressant drugs (ADs) are available, but despite advances in the treatment of MDD, it has been estimated that almost 40% of patients are resistant to conventional treatments. Treatment-resistant depression (TRD) is diagnosed when treatment with two ADs of the different pharmacological profiles, used for a sufficient length of time at an adequate dose, does not improve the patient’s health condition [2]. The mechanisms underlying TRD are not clear and are difficult to study. Nevertheless, one can use Wistar Kyoto (WKY) rats, the strain that has been approved as an appropriate animal model used in preclinical studies on TRD [3, 4]. The WKY strain was isolated in the 1970s of the last century due to inbreeding from the Wistar (WIS) strain, with pressure towards spontaneous hypertension [5]. Then, this strain showed many parameters identical to the “depressive state” observed in other models used in the studies of depression, and the WKY strain has been regarded as a genetic animal model for this disease. WKY rats exhibit outstanding sensitivity to stress as well as a range of characteristic depressive behaviors, such as increased emotionality in a novel environment, an anxiogenic profile on the elevated plus-maze, and increased immobility in the forced swim test, that are not related to stress exposure. This evidence is consistent with the two criteria for a model of TRD. Interestingly, the WKY strain is unresponsive to ADs administration when subjected to a chronic mild stress (CMS) procedure [4, 6, 7], except for deep brain stimulation (DBS) and ketamine (KET, the drug recently widely studied in the context of its antidepressant activity) [8]. The antidepressant effect of KET in WKY rats is especially interesting in the context of data obtained in human studies, showing the rapid and sustained effect of KET given to TRD patients [9]. As the brain region is involved in the effects of KET, lateral division of the habenula (LHb) has been postulated [10, 11]. The mammalian habenula is a small brain region, and it consists of medial (MHb) and lateral (LHb) divisions; these divisions have different patterns of afferent and efferent connections and can be modulated by distinct gene expression profiles. Specifically, the MHb mainly projects to the interpeduncular nucleus, whereas the primary output regions from LHb are midbrain aminergic centers [12]. The habenula has been shown to link the first limbic forebrain structures with monoaminergic nuclei. Regarding efferents, the MHb and LHb receive most of their telencephalic inputs via the fiber tract of the stria medullaris [13]. The MHb has been very intensively studied in the reward circuitry, mediating primarily the aversive properties of distinct drugs of abuse [13–15]. Our previous studies indicated MHb as a stress-sensitive brain region [16–18]. Recently, an interesting new concept has emerged indicating the correlation of depression with excessive activation of this brain region. DBS within the LHb resulted in the reduction of depressive states and increased secretion of aminergic neurotransmitters in the hippocampus and blood [19]. Lesions or DBS of the LHb was reported to ameliorate depression-like symptoms in animal models and treatment-resistant human patients [11, 20, 21]. Comparative fMRI studies of patients responding to antidepressant therapy and TRD patients showed changes in abnormal resting state connectivity in the habenular circuitry. Moreover, the authors provide evidence that, unlike patients responding to ADs, TRD patients are characterized by hyperconnectivity of the left habenula particularly with regions of the default mode network. An increased interplay between reward and default mode networks is linked to suicidality and could be a possible mechanism for anhedonia in hard-to-treat depression [22]. Therefore, it seems that the therapy based on inhibition of habenula hyperactivity may have a significant impact on the success in the treatment of TRD. Considering these results, further research on habenular nuclei in the context of their involvement in TRD is very promising.

Recently, it has been hypothesized that microRNAs (miRNAs) have a role in neuropsychiatric disorders since they are involved in neuropathology-associated processes, such as brain development, dendritic spine morphology, and neurite outgrowth. miRNAs, which are widely expressed in eukaryotes, are small (17–24 nucleotides), noncoding RNA transcripts that play an important role in the posttranscriptional regulation of many genes. miRNA binds to the 3′UTR of targeted mRNA and causes its degradation by recruiting RISC protein complexes. Because of the impressive stability of circulating miRNAs in the bloodstream and their often specific tissue origin, miRNAs serve as potential noninvasive biomarkers of many diseases, including MDD [23–25].

**Fig. 1:**
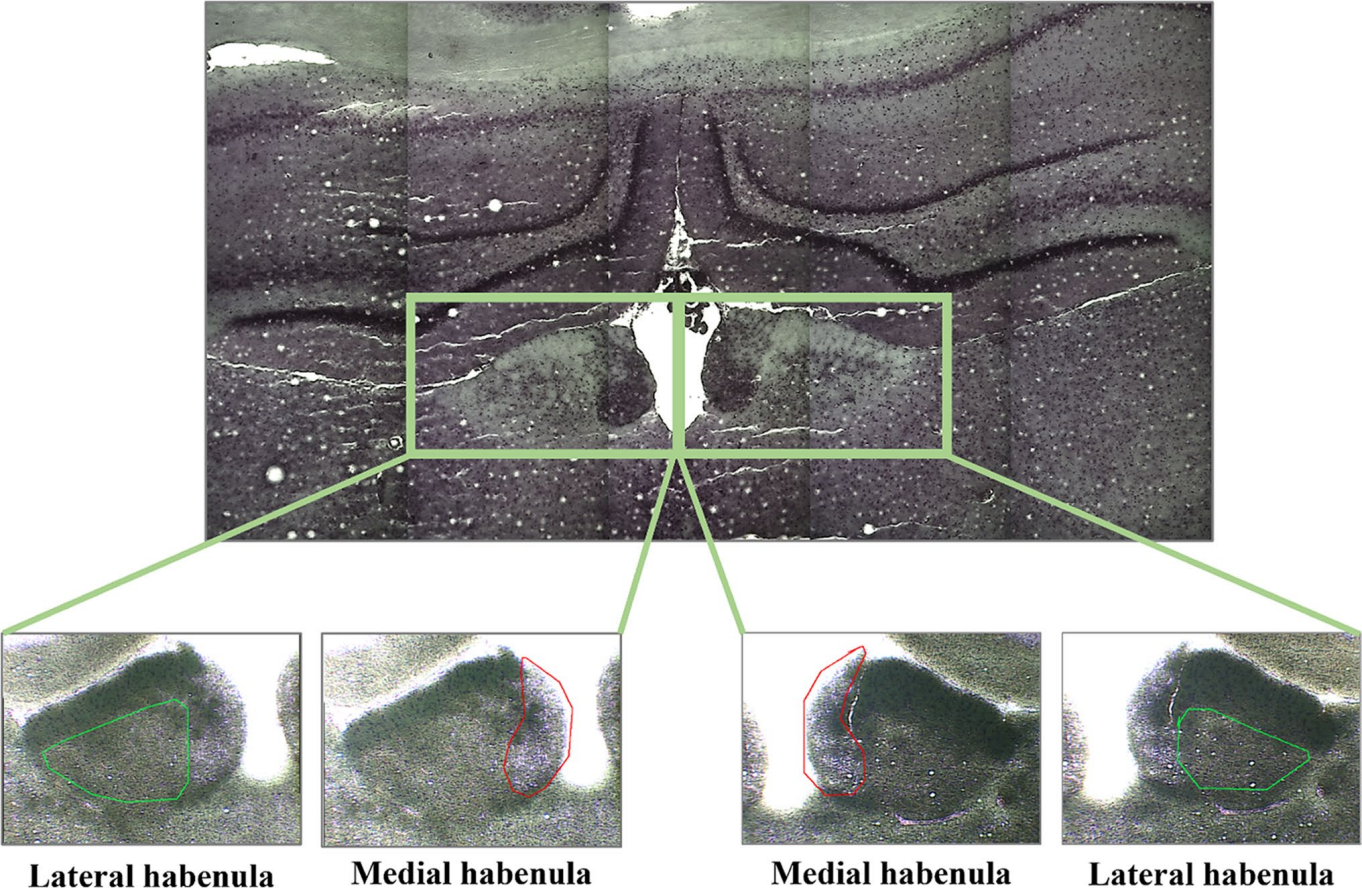
Example of a brain slice stained with cresyl violet. The medial and lateral parts of the habenula, which were dissected using laser microdissection, are highlighted in the images

Therefore, in the first stage of our study, we focused on selecting miRNAs that differentiate the WKY strain from Wistar Han rats (WIS strain) in two habenula nuclei, LHb and MHb (Fig. 1). Based on our preliminary study and literature survey, we identified 32 miRNAs that could be potentially regulated in the habenula. Then, using the miRwalk and TargetScan databases, we selected relevant genes regulated by significant miRNAs, and their expression in the habenular nuclei was investigated.

## Results

### miRNAs Expression Changes in the Medial and Lateral Habenula

**Fig. 2:**
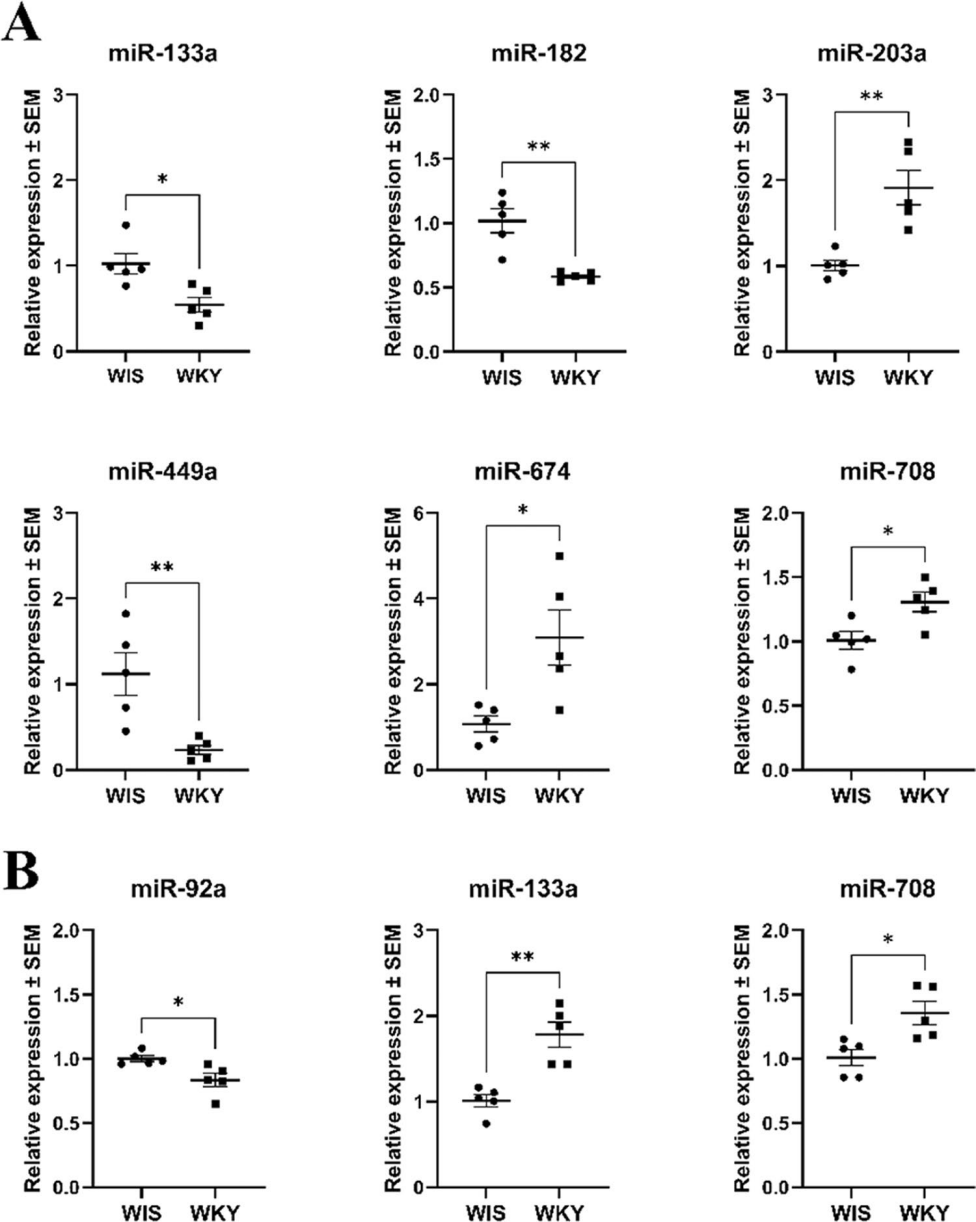
The expression of miRNAs in the medial habenula (MHb) (**A**) and lateral habenula (LHb) (**B**). The results were obtained for *n* = 4–5 and are presented in the graph as miRNA expression fold changes ± SEM. *P* values were estimated using the unpaired *t *test; *p < 0.05, **p < 0.005. WIS, Wistar Han rats; WKY, Wistar Kyoto rats. Since all miRNAs were tested on a single plate, we performed additional statistical analysis with false discovery rate approach using two-stage step method of Benjamin, Krieger, and Yekutieli (FDR < 0.05)

Taking advantage of our preliminary studies, we selected 32 miRNAs important for MDD, and Custom TaqMan Array Cards allowed us to assess their expression in two divisions of the rat habenula. The list of 32 investigated miRNAs is provided in Supplementary Table 1. Most of these miRNAs were expressed at a similar level in both habenula nuclei; however, 6 miRNAs significantly differentiated WKY from WIS rats within MHb (Fig. 2A), 4 of them were specific for MHb, and 2 others were common for both habenular nuclei. The expression levels of 3 of them were lower in WKY rats than in WIS rats: miR-133a (*p* = 0.0121), miR-182 (*p* = 0.0017), miR-449a (*p* = 0.0078); and the other 3 were higher: miR-203a (*p* = 0.0026), miR-674 (*p* = 0.0161), miR-708 (*p* = 0.0182) (Fig. 2A).

In the LHb, 3 miRNAs significantly differentiated WKY from WIS rats (Fig. 2B). The expression levels of 2 of them were higher in WKY rats than in WIS rats: miR-133a (*p* = 0.0016) and miR-708 (*p* = 0.0128); whereas miR-92a (*p* = 0.0191) displayed lower expression (Fig. 2B).

**Fig. 3:**
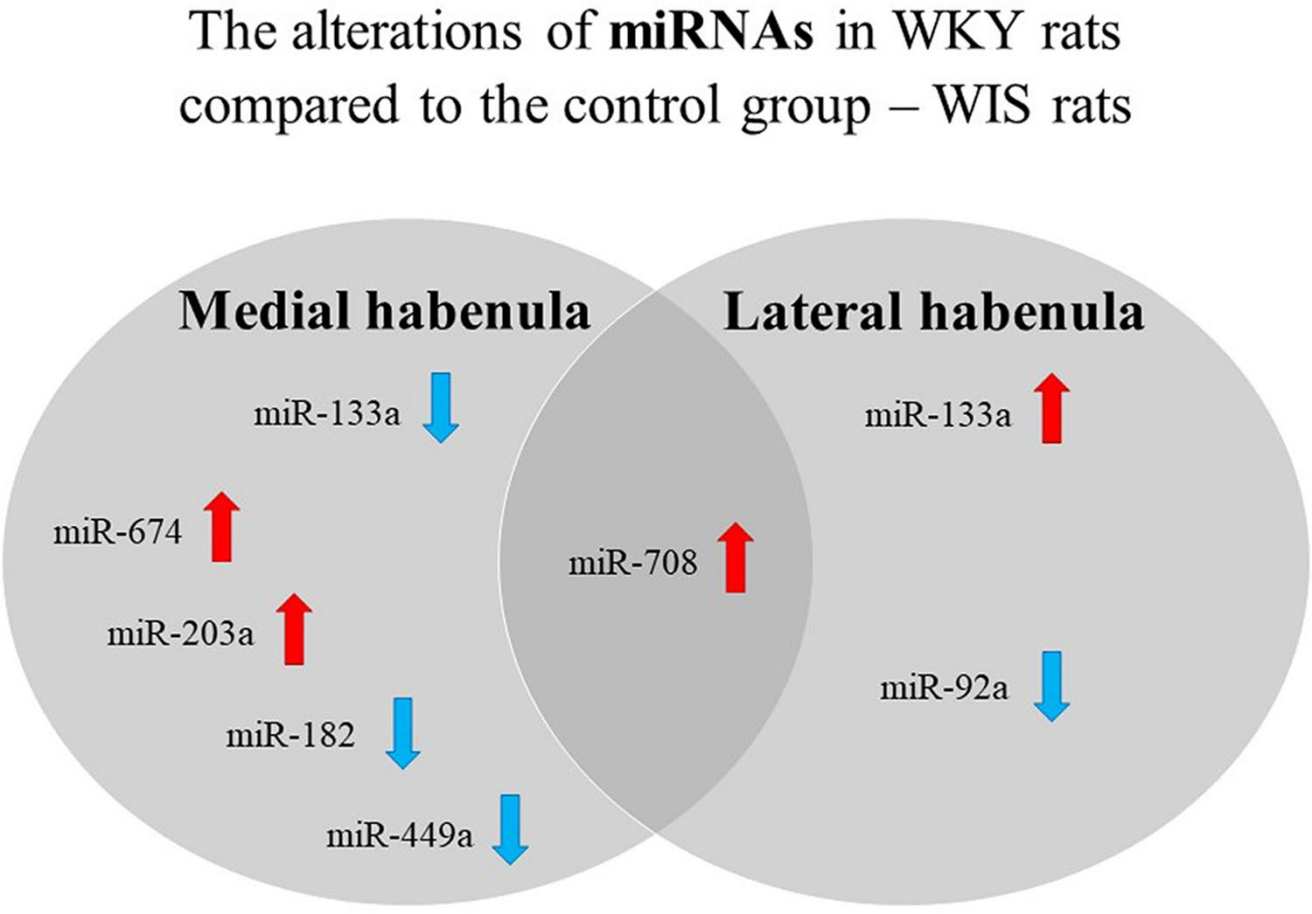
The Venn diagram presents a comparison of miRNA alterations within the MHb and LHb in WKY rats compared to the control group — WIS rats

The obtained results are also presented by the Venn diagram to compare differences in miRNA alterations within MHb and LHb. miRNAs common for both MHb and LHb are miR-133a and miR-708. The expression level of miR-708 was higher in WKY rats than in WIS rats (Fig. 3). Equally interesting is the fact that the expression of miR-133a within the medial habenula is lower in WKY rats, while it is significantly higher within the LHb (Fig. 2A, B ; Fig. 3).

### mRNAs Expression Changes in the Medial and Lateral Habenula

Based on the available databases (miRWalk and Target Scan, open-source platforms that generate predicted and verified miRNA binding sites), we selected potential targets, i.e., mRNAs potentially regulated by miRNAs, which differentiated WKY rats from WIS rats.

**Fig. 4:**
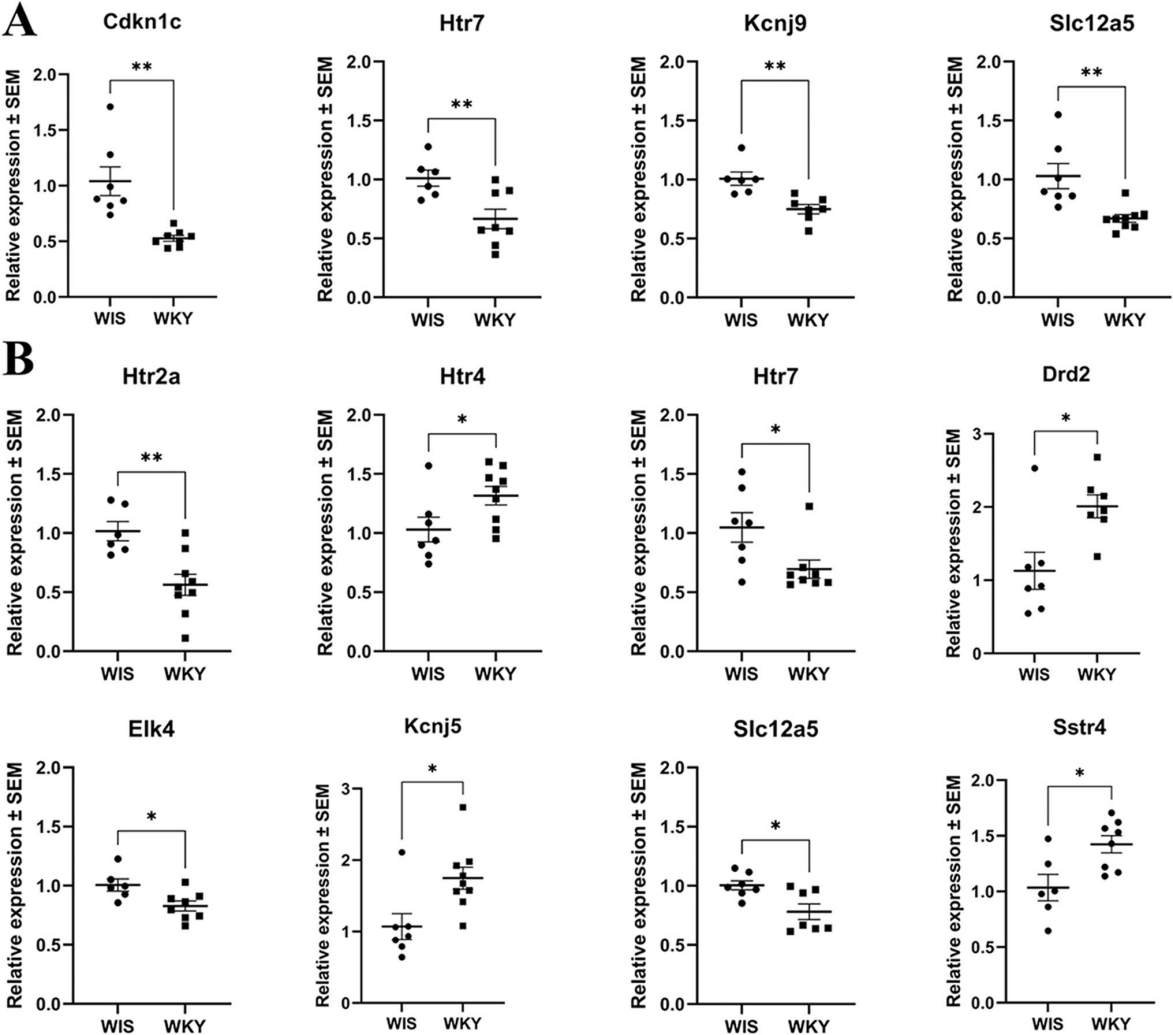
The expression of mRNAs in the medial habenula (**A**) and lateral habenula (**B**). The results were obtained for *n* = 6–9 and are presented in the graph as mRNA expression fold changes ± SEM. *P* values were estimated using the unpaired *t* test; *p < 0.05, **p < 0.01. WIS, Wistar Han rats; WKY, Wistar Kyoto rats

Upon investigation of the expression of 32 mRNAs (Supplementary Table 2), we identified 4 mRNAs that significantly differentiated the WKY strain from WIS within the MHb. The expression levels of all of these genes were lower in WKY rats than in WIS rats: *Cdkn1c* (*p* = 0.0011), *Htr7* (*p* = 0.0096), *Kcnj9* (*p* = 0.0029), and *Slc12a5* (*p* = 0.0028) (Fig. 4A).

On the other hand, 8 mRNAs significantly differentiated WKY rats from WIS rats within the LHb. The expression level of 4 of them was higher in WKY rats in comparison to WIS rats: *Htr4* (*p* = 0.0428),* Drd2* (*p* = 0.0121),* Kcnj5* (*p* = 0.0126),* Sstr4* (*p* = 0.0144); and of the other 4 — lower: *Htr2a* (*p* = 0.0037),* Htr7* (*p* = 0.0288),* Elk4* (*p* = 0.0190),* Slc12a5* (*p* = 0.0129) (Fig. 4B).

**Fig. 5:**
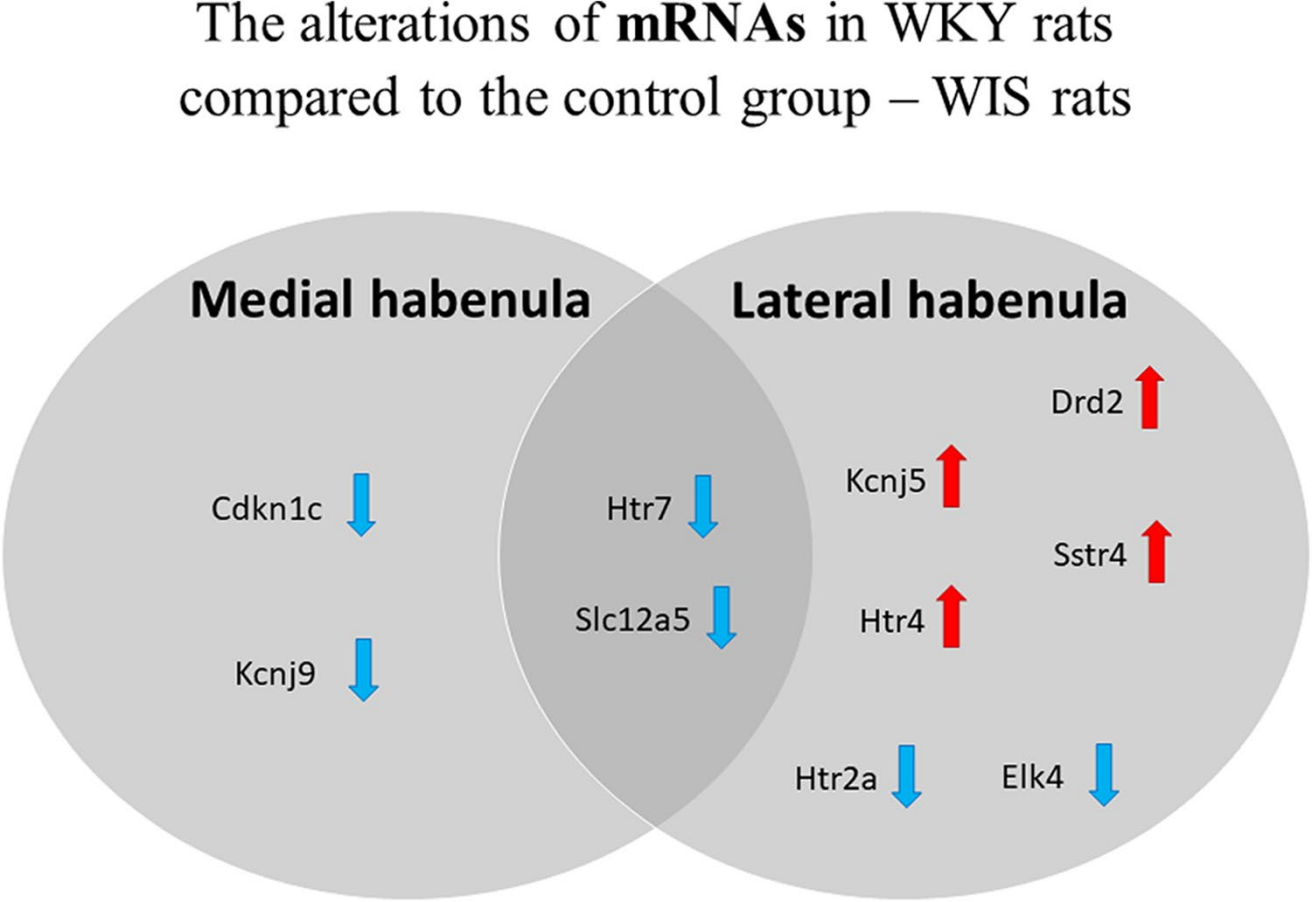
The Venn diagram presents a comparison of mRNA alterations within the MHb and LHb in WKY rats compared to the control group — WIS rats

The obtained results are also presented by the Venn diagram (Fig. 5) to compare differences in mRNA alterations within MHb and LHb. Common mRNAs differentiating the two strains of rats in both MHb and LHb were *Htr7* and *Slc12a5*, and their expression levels were lower in WKY rats than in WIS rats (Fig. 5).

Summing up the obtained results, it is worth noting that slightly more miRNAs were altered in the MHb, but at the mRNA level, more differences between WKY and WIS rats were observed in the LHb. It is interesting in the context of the data provided by Kim and Chang [26], who have shown that the connection between the two habenular nuclei is asymmetrical in that only the medial habenula sends projection to the lateral habenula.

### Immunohistochemistry of 5-HT7 and KCC2

**Fig. 6:**
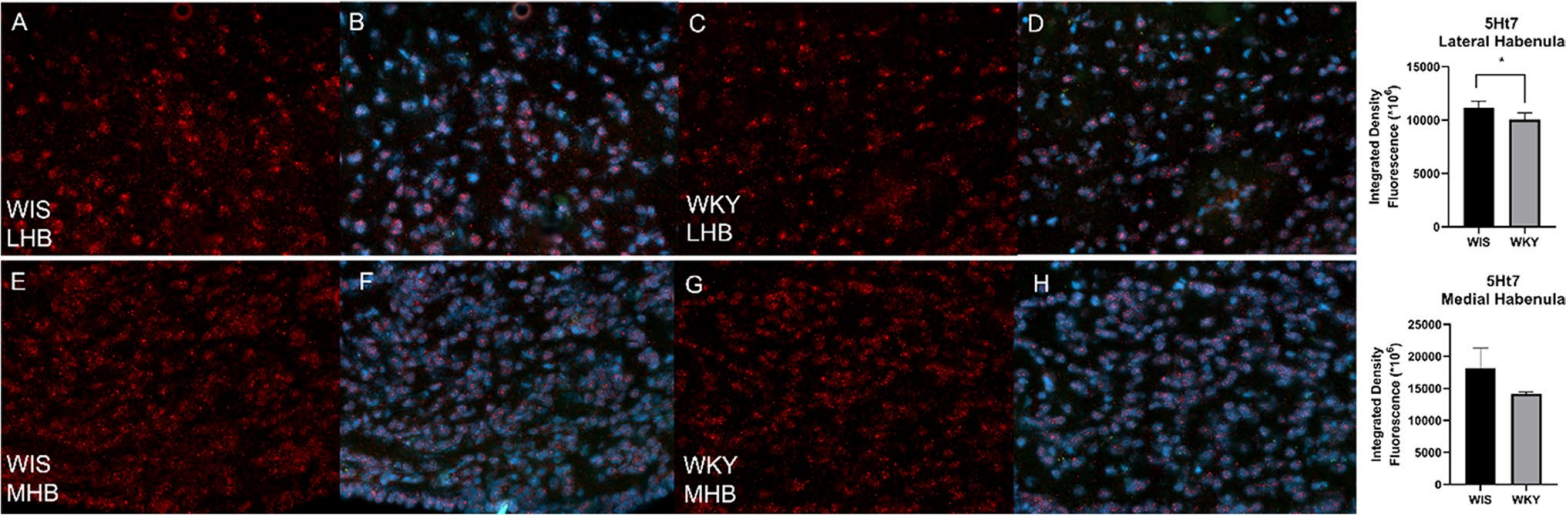
Digital images showing serotonin 5-HT7 receptor expression in the LHb of WIS (**A**, **B**) and WKY (**C**, **D**) and MHb of WIS (**E**, **F**) and WKY (**G**, **H**) rats. Quantification of the integrated density of 5-HT7 receptor reveals decrease of 5-HT7 in LHb (*p < 0.05). Data are presented as means ± SEM. *n* = 4–5 animals per group. Scale bar = 20 μm

**Fig. 7:**
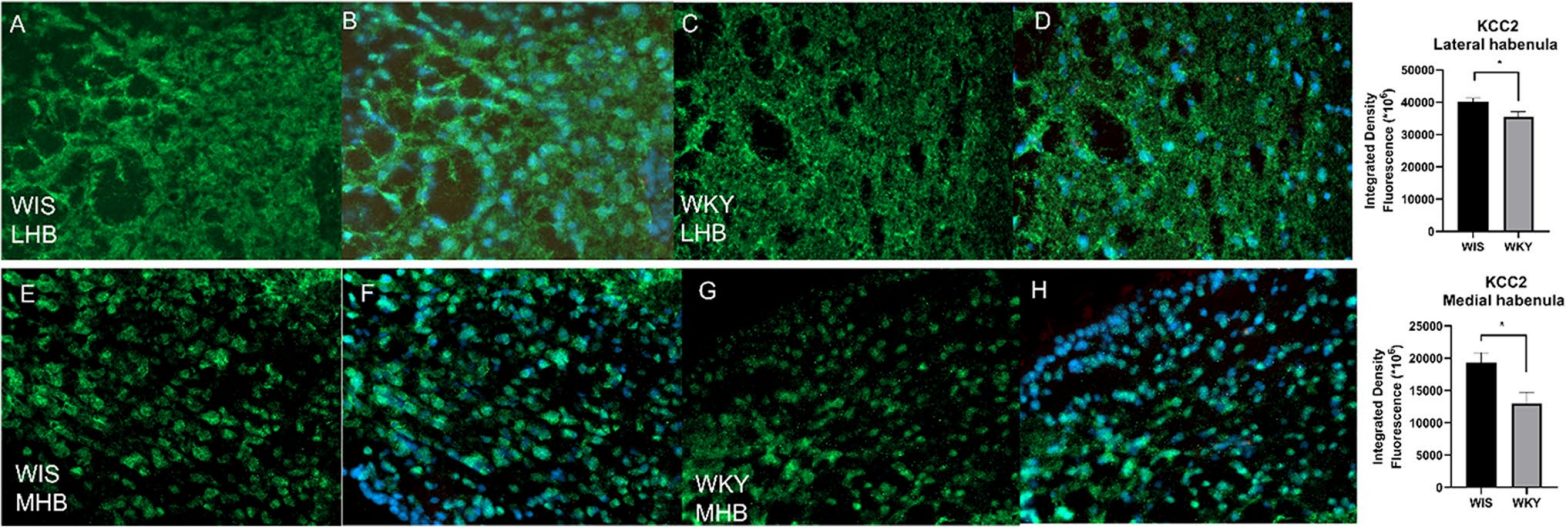
Digital images showing KCC2 expression in the LHb of WIS (**A**, **B**) and WKY (**C**, **D**) and MHb of WIS (**E**, **F**) and WKY (**G**, **H**) rats. Quantification of the integrated density of KCC2 reveals decrease of KCC2 in LHb and MHb (*p < 0.05). Data are presented as means ± SEM. *n* = 4–5 animals per group. Scale bar = 20 μm

Out of the genes for which we observed altered expression, only two were changed in both habenula nuclei. Therefore, we decided to determine the level of proteins encoded by these transcripts. We observed a decrease in the density of serotonin 5-HT7 receptor; however only in LHb, it was statistically significant (*p* = 0.0288) (Fig. 6). KCC2 channel, encoded by the *Slc12a5* gene, was also significantly decreased, both in the LHb (*p* = 0.0432) and MHb (*p* = 0.0328) (Fig. 7).

## Discussion

In the present study, we aimed to search for markers of TRD using WKY rats, which are a well-established animal genetic model of treatment resistant depression. Several studies, mainly based on acute administration of ADs and the forced swim test, indicated that this strain is resistant to antidepressant treatment [6, 27, 28]. Additionally, when subjected to a chronic mild stress procedure, WKY rats did not respond to long-term treatment with various ADs other than subchronic administration of KET or acute DBS of the prefrontal cortex [8, 29, 30], which additionally confirms that this strain is a well-validated TRD model [4]. In the present research, we focused on the habenular nuclei. Most studies published thus far have focused on the lateral division of the habenula. LHb neurons show a significant increase in burst activity and theta-band synchronization in depressive-like animals, which was reversed by KET. Studies using pharmacology and modeling experiments reveal that LHb bursting requires both NMDARs and low-voltage-sensitive T-type calcium channels (T-VSCCs) [11]. In the present work, we investigated both LHb and MHb to find possible differences between WKY and WIS rats at the level of their miRNAs content. As a result of our preliminary research and a literature survey, we identified 32 miRNAs that could be potentially regulated in the habenula. Since the MHb and LHb nuclei differ not only in anatomical demarcation but also in projections and gene expression, we decided to look at these two nuclei separately. Out of 32 miRNAs, 7 miRNAs changed significantly.

Among miRNAs that differentiated the WKY strain from WIS, miR-92a deserves attention, since it regulates the expression of a few interesting transcripts. In the present study, we observed downregulation of miR-92a in the LHb of WKY rats, which correlated with an increase in *Kcnj5* mRNA expression in this brain region (the *Kcnj5* gene encodes for potassium inwardly rectifying channel subfamily J member 5, Girk4). Several pharmacogenomic studies have examined Girk4 gene variants concerning antidepressant treatment outcomes [31, 32]. Girk4 encodes the kainic acid-type glutamate receptor 1 (KA1) subunit, which coassembles with other glutamate receptor subunits to form cation-selective ion channels. Moreover, it can perform metabotropic functions [33, 34]. An antidepressant phenotype associated with ablation of Girk4 has been recently demonstrated [35]. In the context of these data, the increase in Girk4 mRNA expression observed in the LHb of the WKY rats in the present study indicates the potential role of this channel in the mechanisms underlying TRD.

miR-92a is also involved in the regulation of the transcript encoding the serotonin 5-HT4 receptor, and its downregulation observed in the LHb of WKY rats correlates with upregulation of 5-HT4 mRNA expression. Recently, it has been shown that activation or blockade of the 5-HT4 receptors in the LHb produces antidepressant effects in 6-OHDA-lesioned rats, which are related to changes in monoamines in limbic and limbic-related regions [36]. Increased biosynthesis of this receptor in WKY rats might also be interpreted as contributing to their depressive phenotype — especially in the context of studies by Vidal et al. [37, 38], who have shown that chronic administration of monoaminergic antidepressants, such as fluoxetine, paroxetine, and venlafaxine (but not reboxetine), decreased 5-HT4 receptor density in the rat brain.

Another transcript regulated by miR-92a was mRNA encoding somatostatin receptor type 4 (Sst4); we observed increased expression of this transcript in the LHb of WKY rats. In our previous study, we showed that the Sst4 receptor is involved in the stress response of WIS rats subjected to chronic mild stress, although changes were observed in the MHb [17]. Additionally, it has been shown that mice with Sst4 knockout showed increased anxiety and depression-like behavior, while J-2156 (a Sstr4 agonist) exerted anxiolytic- and antidepressant-like effects [39, 40]. These data appear to be in opposition to the results we obtained and require further explanation.

The common nature of the changes was observed for both habenula divisions for one miRNA: miR-708. Despite the statistical trends, the multiple *t* tests did not show significant changes within miR-203a in LHb. However, it was observed to change significantly statistically within MHb. Both miRNAs, miR-708 and miR-203a, regulate the mRNA encoding *Slc12a5*. As we have shown, upregulation of miR-203a and miR-708 in WKY rats correlated with downregulation of *Slc12a5* mRNA expression. The *Slc12a5* gene encodes the neuronal KCC2 channel, which is the major extruder of intracellular chloride in mature neurons. In the presence of low intraneuronal chloride, the binding of GABA and glycine to their ionotropic receptors results in chloride influx with subsequent hyperpolarization contributing to neuronal inhibition. KCC2 dysregulation has been associated with numerous neurological and psychiatric disorders. In most cases, reduced KCC2 expression was associated with depolarized GABAAR-mediated currents [41]. It has been shown that stress can also directly affect the regulation of KCC2. In adult mice, repeated stress can lead to KCC2 downregulation [42, 43]. After chronic restraint stress (30 min/day for 14 consecutive days) but not acute stress, dephosphorylation of KCC2 at the S940 residue was observed, which might explain KCC2 downregulation [44]. Since stress is one of the major causes of dysregulation of the hypothalamic–pituitary–adrenal axis and KCC2 has been shown to play a role in GABAergic regulation of corticotropin releasing hormone (CRH) neurons in the paraventricular nucleus of the hypothalamus, which is critical for triggering the body’s physiological stress response [45, 46], it seems that the role of KCC2 may be important in depression. Downregulation of mRNA encoding KCC2 as well as the protein level in both habenula divisions of WKY rats might contribute to their depressive phenotype.

A nother interesting finding of the present study points to miR-708, miR-674, and miR-133a, whose increased expression was observed in the habenula. These miRNAs play important roles in the regulation of mRNA encoding the serotonin 5-HT7 receptor. The involvement of serotonin receptors in the pathogenesis of depression and antidepressant effects has been widely described. The earliest studies using 5-HT7 receptor knockout mice showed a significant decrease in immobility compared to controls, a phenotype similar to antidepressant-treated mice in the forced swim test [47]. Subsequently, SB-269970 reduced immobility time in the FST test without modifying locomotor activity [48]. Recently, the role of 5-HT7 receptor antagonists in antidepressant effects has been raised. Moreover, pharmacological blockade of the serotonin 5-HT7 receptor produces a faster antidepressant effect than the commonly used drug fluoxetine [49]. In the present work, we observed a decrease in mRNA encoding this receptor in both habenular nuclei, which indicates its lower biosynthesis. In addition, we also observed a decrease in 5-HT7 levels in LHb. Little is known about 5-HT7R agonists; however, there are studies indicating that 5-HT7R agonists have anxiolytic-like effects [50, 51].

Also, there is no information about the interaction of KCC2 and 5-HT7R in habenula; however, there are reports indicating that the administration of 5-HT2AR and 5-HT7R agonists upregulates KCC2, thereby restoring endogenous inhibition and reducing spasticity in spinal cord injury [52]. Serotonin 5-HT2AR agonists have been shown to affect KCC2 phosphorylation. It has been shown that stress-induced inhibitory plasticity circuitry was reversible after treatment with the 5-HT2AR agonist TCB-2 ex vivo by functionally enhancing the KCC2. The signaling pathway linking 5-HT2AR activation and normalization of KCC2 function was dependent on protein kinase C signaling and KCC2 phosphorylation at serine 940 (S940) because mutation of S940 to alanine prevented TCB-2 from restoring chloride transporter function [53]. Furthermore, it has been shown that the activation of 5-HT2AR hyperpolarizes the reversal potential of inhibitory postsynaptic potentials (IPSPs). IPSPs in spinal motoneurons increase the cell membrane expression of KCC2 and both restore endogenous inhibition. Therefore, the regulation of KCC2 expression and function by targeting 5-HT2AR has therapeutic potential in the treatment of neurological disorders involving altered chloride homeostasis [52]. Thus, it appears that activation of 5-HT7 receptors may have antidepressant potential in TRD, acting to upregulate KCC2 and restore endogenous inhibition in the habenula, which is overstimulated in TRD.

### Limitation

In the present work, we mainly focus on studying the expression levels of miRNAs, their mRNA targets, and the selected proteins in the habenular nuclei, which are tiny brain regions. There are technical limitations in obtaining sufficient amount of material to measure miRNAs, mRNAs, and proteins (for Western blotting) from the same sample. To overcome these difficulties, we performed immunohistochemical analysis to examine the levels of 5-HT7R and KCC2 in the rat brain slices containing habenular nuclei. The results are provided as integrated density, in which — although frequently used — it is not a perfect tool for the quantification of the level of protein of interest.

## Conclusion

The obtained results confirm that the WKY strain of rats is an interesting research model in the context of TRD. The data described above indicate that several important miRNAs are expressed in the habenula, which differentiates WKY rats from WIS rats. The changes in the expression of these miRNAs correlate with alterations in the expression of mRNAs selected as their appropriate targets. The approach employed in the present work, i.e., starting from the studies at the level of miRNAs as biomarkers in an animal model of TRD and then evaluating the expression of their potential targets (mRNAs), allowed us to identify a few important transcripts that might be responsible for the depressive phenotype exhibited by WKY rats. In this context, the KCC2 channel and serotonin 5-HT7 receptor deserve special attention; however, further detailed studies are necessary. Nevertheless, the present work points to the nuclei of the habenula as a potential target of research on the pharmacotherapy of TRD.

## Materials and Methods

### Animals

Male Wistar Han rats and male Wistar Kyoto rats, at the age of 7 weeks and weighing approximately 180–210 g, were acquired from Charles River (Sulzfeld, Germany). Rats were housed in five animals per standard laboratory cage with free access to food and water and maintained on a 12-h light/dark cycle (lights on at 8 am) under conditions of constant temperature (22 ± 2 °C) and humidity (45 ± 5%). This study was approved by the II Ethical Committee at the Maj Institute of Pharmacology at the Polish Academy of Sciences, Kraków, Poland.

### Tissue Preparation

The brains were isolated from decapitated animals, rapidly frozen using isopentane on dry ice, and stored at −80 °C until further stages. Afterwards, the brain Sects. (20 μm) containing the region of interest — the habenula — were cut using a Jung CM 3000 cryostat microtome (Leica, Wetzlar, Germany) according to Rat Brain Atlas [54]. The slices were then attached to PEN Membrane Glass Slides 2.0 µm (ThermoFisher Scientific, Waltham, MA, USA) and stained with Cresyl Violet from the LCM Staining Kit (Life Technologies, Carlsbad, CA, USA) according to the manufacturer’s instructions. Using laser capture microdissection (Leica, Wetzlar, Germany), the medial and lateral habenular nuclei were obtained.

### Isolation of MicroRNA from Medial and Lateral Habenula Nuclei

miRNA purification was carried out using a miRNeasy Micro Kit (Qiagen, Hilden, Germany) according to the manufacturer’s instructions. The quality and quantity of the isolated miRNA were assessed using a NanoDrop ND-1000 (Thermo Fisher Scientific, Waltham, MA, USA).

### miRNA RT–qPCR Array

According to our preliminary study, custom TaqMan Array MicroRNA Cards (Thermo Fisher Scientific, Waltham, MA, USA) containing 32 selected primers were designed. Samples were duplicated, and one Custom TaqMan Array MicroRNA Card was used per 4 samples running in triplicate. Reverse transcription reactions were conducted with a TaqMan MicroRNA Reverse Transcription Kit (Thermo Fisher Scientific, Waltham, MA, USA) according to the manufacturer’s instructions. The obtained cDNA was preamplified to increase the quantity of cDNA using TaqMan PreAmp Master Mix (Thermo Fisher Scientific, Waltham, MA, USA) according to the manufacturer’s instructions. The preamplified product was diluted, and qPCR was performed using TaqMan Universal PCR Master Mix, No AmpErase UNG (Thermo Fisher Scientific, Waltham, MA, USA). The qPCRs were run on a QuantStudio 12 K Flex System (Applied Biosystems, Waltham, MA, USA).

Data were analyzed with the QuantStudio 12 K Flex Software (Applied Biosystems, Waltham, MA, USA). The same threshold equal to 0.2 was set for all samples for comparison. A Ct value above 31 was considered undetectable miRNAs due to performing a preamplification reaction. Then, qbase^+^ by the Biogazelle software (Biogazelle, Zwijnaarde, Belgium) was used, and the geNorm algorithm-generated miRNAs, miR-7a* and miR-328, were suitable for normalization [55, 56]. Subsequently, the relative expression level was calculated with the 2^−ΔΔCT^ method according to Pfaffl (2001) [57] with a modified equation for multiple references. Statistical analysis was carried out with GraphPad Prism 9.1.2 by an unpaired *t *test. A value of *p* < 0.05 was considered statistically significant.

### Isolation of mRNA from Medial and Lateral Habenular Nuclei

mRNA purification was carried out using an RNAeasy Micro Kit (Qiagen, Hilden, Germany) according to the manufacturer’s instructions. The total RNA concentration was measured with a NanoDrop ND-1000 Spectrometer (Thermo Fisher Scientific, Waltham, MA, USA), and the RNA quality was assessed by microcapillary electrophoresis using an Experion RNA HighSens Analysis Kit (Bio–Rad, Hercules, CA, USA) according to the manufacturer’s instructions. For further experiments, samples that passed the quality threshold (RIN > 8.0) were used.

### mRNA RT–qPCR Array

According to the results obtained from miRNA analysis and the miRWalk and Target Scan databases, appropriate Custom TaqMan Gene Expression Array Cards (Thermo Fisher Scientific, Waltham, MA, USA) with 32 selected primers were designed to determine the expression level of the mRNAs. One Custom TaqMan Gene Expression Array Card was used per 4 samples running in triplicate. Reverse transcription reactions were conducted with a High-Capacity cDNA Reverse Transcription Kit (Thermo Fisher Scientific, Waltham, MA, USA) according to the manufacturer’s instructions. The qPCR assays were performed using a TaqMan Universal PCR Master Mix, No AmpErase UNG (Thermo Fisher Scientific, Waltham, MA, USA) according to the manufacturer’s instructions and were run on a QuantStudio 12 K Flex System (Applied Biosystems, Waltham, MA, USA).

Data were analyzed with the QuantStudio 12 K Flex Software (Applied Biosystems, Waltham, MA, USA). The same threshold equal to 0.2 was set for all samples for comparison. A Ct value above 34 was considered undetectable mRNAs. Subsequently, the relative gene expression level was calculated with the 2^−ΔΔCT^ method according to Pfaffl (2001) [57] with a modified equation for multiple reference genes: ribosomal protein L32 (*Rpl32*), peptidylprolyl isomerase A (*Ppia*), and eukaryotic 18S ribosomal RNA (*18S*). Statistical analysis was carried out with GraphPad Prism 9.1.2 by an unpaired *t *test. A value of *p* < 0.05 was considered statistically significant.

### Immunohistochemistry

Frozen rat brains were cut using a cryostat microtome to obtain slices containing the habenula according to Rat Brain Atlas. Brain slices (16 µm) were placed on glass slides, fixed for 10 min in ice-cold methanol, washed in 0.1 M PBS three times for 10 min, and then blocked in 10% normal donkey serum (NDS) in 0.1 M PBS overnight at 4 °C. The following day, brain slices were incubated with the primary antibody in 5% NDS in 0.1 M PBS (5-HT7 1:100, #ABIN617922, antibodies-online; KCC2, 1:200, #07–432, Sigma Aldrich) overnight at 4 °C. After three washes in 0.1 M PBS for 10 min, brain slices were incubated with anti-rabbit Alexa488 (1:200, #A21206, Invitrogen) or anti-rabbit Alexa555 (1:200, #A31572, Invitrogen) secondary antibodies for 2 h in RT. Slides were washed again three times with 0.1 M PBS and stained with 4,6-diamidino-2-phenylindole (DAPI) to visualize nuclei. Slides were covered and slipped with VECTASHIELD Antifade Mounting Medium (Vector Laboratories). Images were acquired using the Axio Imager A2 fluorescence microscope (Carl Zeiss, Germany). The integrated density was measured with ImageJ.

### Statistical Analysis

Data analysis included the identification of outliers and assessment of the normal distribution using the Shapiro–Wilk test and the Kolmogorov–Smirnov test. A significance level (alpha level) of 5% was used.

Statistical analysis results of all data were compared using an unpaired *t *test to assess differentiation between WIS and WKY rat strains. Since the expression were measured simultaneously on one plate, a statistical analysis was additionally performed based on the False Discovery Rate approach using the two-stage step method of Benjamin, Krieger, and Yekutieli (FDR < 0.05). The analysis was carried out using GraphPad Prism 9.1.2.

## Supplementary Information

Below is the link to the electronic supplementary material.

Supplementary file1 (DOCX 19 KB)

Supplementary file2 (DOCX 21 KB)

## Data Availability

The datasets generated and analyzed during the current study are available in the Institute of Pharmacology Polish Academy of Sciences in the Biochemical Pharmacology Laboratory and can be available upon individual request with the corresponding author.

## References

[1] Otte C, Gold SM, Penninx BW (2016). Major depressive disorder. Nat Rev Dis Primers.

[2] Jaffe DH, Rive B, Denee TR (2019). The humanistic and economic burden of treatment-resistant depression in Europe: a cross-sectional study. BMC Psychiatry.

[3] Millard SJ, Weston-Green K, Newell KA (2020). The Wistar-Kyoto rat model of endogenous depression: A tool for exploring treatment resistance with an urgent need to focus on sex differences. Prog Neuropsychopharmacol Biol Psychiatry.

[4] Aleksandrova LR, Wang YT, Phillips AG (2019). Evaluation of the Wistar-Kyoto rat model of depression and the role of synaptic plasticity in depression and antidepressant response. Neurosci Biobehav Rev.

[5] Gentsch C, Lichtsteiner M, Feer H (1988). Genetic and environmental influences on behavioral and neurochemical aspects of emotionality in rats. Experientia.

[6] Lahmame A, del Arco C, Pazos A (1997). Are Wistar-Kyoto rats a genetic animal model of depression resistant to antidepressants?. Eur J Pharmacol.

[7] Papp M, Gruca P, Faron-Górecka A (2019). Genomic Screening of Wistar and Wistar-Kyoto Rats Exposed to Chronic Mild Stress and Deep Brain Stimulation of Prefrontal Cortex. Neuroscience.

[8] Willner P, Gruca P, Lason M (2019). Validation of chronic mild stress in the Wistar-Kyoto rat as an animal model of treatment-resistant depression. Behav Pharmacol.

[9] Zarate CA, Singh JB, Carlson PJ (2006). A randomized trial of an N-methyl-D-aspartate antagonist in treatment-resistant major depression. Arch Gen Psychiatry.

[10] Gold PW, Kadriu B (2019). A Major Role for the Lateral Habenula in Depressive Illness: Physiologic and Molecular Mechanisms. Front Psychiatry.

[11] Yang Y, Cui Y, Sang K (2018). Ketamine blocks bursting in the lateral habenula to rapidly relieve depression. Nature.

[12] Aizawa H, Kobayashi M, Tanaka S (2012). Molecular characterization of the subnuclei in rat habenula. J Comp Neurol.

[13] Bueno D, Lima LB, Souza R (2019). Connections of the laterodorsal tegmental nucleus with the habenular-interpeduncular-raphe system. J Comp Neurol.

[14] Antolin-Fontes B, Ables JL, Görlich A, Ibañez-Tallon I (2015). The habenulo-interpeduncular pathway in nicotine aversion and withdrawal. Neuropharmacology.

[15] Arvin MC, Jin XT, Yan Y (2019). Chronic Nicotine Exposure Alters the Neurophysiology of Habenulo-Interpeduncular Circuitry. J Neurosci.

[16] Faron-Górecka A, Kuśmider M, Kolasa M (2016). Chronic mild stress alters the somatostatin receptors in the rat brain. Psychopharmacology.

[17] Faron-Górecka A, Kuśmider M, Solich J (2018). Behavioral response to imipramine under chronic mild stress corresponds with increase of mRNA encoding somatostatin receptors sst2 and sst4 expression in medial habenular nucleus. Neurochem Int.

[18] Faron-Górecka A, Kuśmider M, Solich J (2018). Regulation of somatostatin receptor 2 in the context of antidepressant treatment response in chronic mild stress in rat. Psychopharmacology.

[19] Dandekar MP, Fenoy AJ, Carvalho AF (2018). Deep brain stimulation for treatment-resistant depression: an integrative review of preclinical and clinical findings and translational implications. Mol Psychiatry.

[20] Sartorius A, Kiening KL, Kirsch P (2010). Remission of major depression under deep brain stimulation of the lateral habenula in a therapy-refractory patient. Biol Psychiatry.

[21] Meng H, Wang Y, Huang M (2011). Chronic deep brain stimulation of the lateral habenula nucleus in a rat model of depression. Brain Res.

[22] Barreiros AR, Breukelaar I, Mayur P (2022). Abnormal habenula functional connectivity characterizes treatment-resistant depression. Neuroimage Clin.

[23] Perelló Ferrúa C, Giorgi R, Da Rosa LC (2019). MicroRNAs expressed in depression and their associated pathways: A systematic review and a bioinformatics analysis. J Chem Neuroanat.

[24] Belzeaux R, Lin R, Turecki G (2017). Potential Use of MicroRNA for Monitoring Therapeutic Response to Antidepressants. CNS Drugs.

[25] Belzeaux R, Bergon A, Jeanjean V (2012). Responder and nonresponder patients exhibit different peripheral transcriptional signatures during major depressive episode. Transl Psychiatry.

[26] Kim U, Chang SY (2005). Dendritic morphology, local circuitry, and intrinsic electrophysiology of neurons in the rat medial and lateral habenular nuclei of the epithalamus. J Comp Neurol.

[27] López-Rubalcava C, Lucki I (2000). Strain differences in the behavioral effects of antidepressant drugs in the rat forced swimming test. Neuropsychopharmacology.

[28] Tejani-Butt S, Kluczynski J, Paré WP (2003). Strain-dependent modification of behavior following antidepressant treatment. Prog Neuropsychopharmacol Biol Psychiatry.

[29] Papp M, Gruca P, Lason M (2020). AMPA receptors mediate the pro-cognitive effects of electrical and optogenetic stimulation of the medial prefrontal cortex in antidepressant non-responsive Wistar-Kyoto rats. J Psychopharmacol.

[30] Papp M, Gruca P, Lason M (2018). Rapid antidepressant effects of deep brain stimulation of the pre-frontal cortex in an animal model of treatment-resistant depression. J Psychopharmacol.

[31] Milanesi E, Bonvicini C, Congiu C (2015). The role of GRIK4 gene in treatment-resistant depression. Genet Res (Camb).

[32] Horstmann S, Binder EB (2009). Pharmacogenomics of antidepressant drugs. Pharmacol Ther.

[33] Nowak G, Li Y, Paul IA (1996). Adaptation of cortical but not hippocampal NMDA receptors after chronic citalopram treatment. Eur J Pharmacol.

[34] Boyer P, Skolnick P, Fossom LH (1998). Chronic administration of imipramine and citalopram alters the expression of NMDA receptor subunit mRNAs in mouse brain. J Mol Neurosci.

[35] Catches JS, Xu J, Contractor A (2012). Genetic ablation of the GluK4 kainate receptor subunit causes anxiolytic and antidepressant-like behavior in mice. Behav Brain Res.

[36] Guo Y, Zhang L, Zhang J (2021). Activation and Blockade of Serotonin-4 Receptors in the Lateral Habenula Produce Antidepressant Effects in the Hemiparkinsonian Rat. Neuropsychobiology.

[37] Vidal R, Castro E, Pilar-Cuéllar F (2014). Serotonin 5-HT4 receptors: A new strategy for developing fast acting antidepressants?. Curr Pharm Des.

[38] Vidal R, Valdizan EM, Vilaró MT (2010). Reduced signal transduction by 5-HT4 receptors after long-term venlafaxine treatment in rats. Br J Pharmacol.

[39] Scheich B, Gaszner B, Kormos V (2016). Somatostatin receptor subtype 4 activation is involved in anxiety and depression-like behavior in mouse models. Neuropharmacology.

[40] Prévôt TD, Gastambide F, Viollet C (2017). Roles of Hippocampal Somatostatin Receptor Subtypes in Stress Response and Emotionality. Neuropsychopharmacology.

[41] Goutierre M, Al Awabdh S, Donneger F (2019). KCC2 Regulates Neuronal Excitability and Hippocampal Activity via Interaction with Task-3 Channels. Cell Rep.

[42] Tsukahara T, Masuhara M, Iwai H (2015). Repeated stress-induced expression pattern alterations of the hippocampal chloride transporters KCC2 and NKCC1 associated with behavioral abnormalities in female mice. Biochem Biophys Res Commun.

[43] Tsukahara T, Masuhara M, Iwai H (2016). The effect of repeated stress on KCC2 and NKCC1 immunoreactivity in the hippocampus of female mice. Data Brief.

[44] MacKenzie G, Maguire J (2015). Chronic stress shifts the GABA reversal potential in the hippocampus and increases seizure susceptibility. Epilepsy Res.

[45] Hewitt SA, Wamsteeker JI, Kurz EU, Bains JS (2009). Altered chloride homeostasis removes synaptic inhibitory constraint of the stress axis. Nat Neurosci.

[46] Sarkar J, Wakefield S, MacKenzie G (2011). Neurosteroidogenesis is required for the physiological response to stress: role of neurosteroid-sensitive GABAA receptors. J Neurosci.

[47] Guscott M, Bristow LJ, Hadingham K (2005). Genetic knockout and pharmacological blockade studies of the 5-HT7 receptor suggest therapeutic potential in depression. Neuropharmacology.

[48] Wesołowska A, Tatarczynska E, Nikiforuk A, Chojnacka-Wojcik E (2007). Enhancement of the anti-immobility action of antidepressants by a selective 5-HT7 receptor antagonist in the forced swimming test in mice. Eur J Pharmacol.

[49] Mnie-Filali O, Faure C, Lambás-Señas (2011). Pharmacological blockade of 5-HT7 receptors as a putative fast acting antidepressant strategy. Neuropsychopharmacology.

[50] Adriani W, Travaglini D, Lacivita E (2012). Modulatory effects of two novel agonists for serotonin receptor 7 on emotion, motivation and circadian rhythm profiles in mice. Neuropharmacology.

[51] De Filippis B, Nativio P, Fabbri A (2014). Pharmacological stimulation of the brain serotonin receptor 7 as a novel therapeutic approach for Rett syndrome. Neuropsychopharmacology.

[52] Gackière F, Vinay L (2014). Serotonergic modulation of postsynaptic inhibition and locomotor alternating pattern in the spinal cord. Front Neural Circuits.

[53] Kimmey BA, Ostroumov A, Dani JA (2019). 5-HT2A receptor activation normalizes stress-induced dysregulation of GABAergic signaling in the ventral tegmental area. Proc Natl Acad Sci U S A.

[54] Paxinos G, Watson C (1998) The rat brain in stereotaxic coordinates, 4th edn. International Standard Book Number: 0–12–547617–5. Academic Press, Cambridge

[55] Vandesompele J, De Preter K, Pattyn F (2002). Accurate normalization of real-time quantitative RT-PCR data by geometric averaging of multiple internal control genes. Genome Biol.

[56] Marabita F, de Candia P, Torri A (2016). Normalization of circulating microRNA expression data obtained by quantitative real-time RT-PCR. Brief Bioinform.

[57] Pfaffl MW (2001). A new mathematical model for relative quantification in real-time RT–PCR. Nucleic Acids Res.

