# Supplementary material for: Habenula as a Possible Target for Treatment-Resistant Depression Phenotype in Wistar Kyoto Rats
Source: Mol Neurobiol. 2022 Nov 8;60(2):643–54. doi: 10.1007/s12035-022-03103-y (PMC9849162; doi:10.1007/s12035-022-03103-y)
Supplement: Supplementary file 1 — Supplementary file1 (DOCX 19 KB) [file 12035_2022_3103_MOESM1_ESM.docx]

Table 1. The table contains information about investigated miRNA and comparisons expression levels in habenular nuclei in two rat strains - WIS (control group) and WKY (experimental group).

| **Assay Name** | **miRBase ID** | **Assay ID** | **miRBase Accession Number** | **Expression level MHb** | **Expression level LHb** |
| --- | --- | --- | --- | --- | --- |
| **hsa-miR-92** | rno-miR-92a-3p | 000430 | MIMAT0000816 | no differentiation | significant statistical difference |
| **hsa-miR-133a** | rno-miR-133a-3p | 002246 | MIMAT0000839 | significant statistical difference | significant statistical difference |
| **mmu-miR-182** | rno-miR-182 | 002599 | MIMAT0005300 | significant statistical difference | beyond the detection limit |
| **hsa-miR-203** | rno-miR-203a-3p | 000507 | MIMAT0000876 | significant statistical difference | significant statistical difference |
| **hsa-miR-449** | rno-miR-449a-5p | 001030 | MIMAT0001543 | significant statistical difference | no differentiation |
| **hsa-miR-674** | rno-miR-674-5p | 002021 | MIMAT0005329 | significant statistical difference | no differentiation |
| **hsa-miR-708** | rno-miR-708-5p | 002341 | MIMAT0005331 | significant statistical difference | significant statistical difference |
| **hsa-miR-184** | rno-miR-184 | 000485 | MIMAT0000861 | no differentiation | no differentiation |
| **U6 snRNA** | - | 001973 | NR_004394 (NCBI Accession Number) | no differentiation | no differentiation |
| **hsa-miR-19a** | rno-miR-19a-3p | 000395 | MIMAT0000789 | no differentiation | no differentiation |
| **ath-miR159a** | ath-miR159a | 000338 | MIMAT0000177 | beyond the detection limit | beyond the detection limit |
| **hsa-miR-29a** | rno-miR-29a-3p | 002112 | MIMAT0000802 | no differentiation | no differentiation |
| **hsa-miR-214** | rno-miR-214-3p | 000517 | MIMAT0000885 | beyond the detection limit | no differentiation |
| **hsa-miR-221** | rno-miR-221-3p | 000524 | MIMAT0000890 | no differentiation | no differentiation |
| **hsa-miR-223** | rno-miR-223-3p | 000526 | MIMAT0000892 | no differentiation | no differentiation |
| **hsa-miR-23a** | rno-miR-23a-3p | 000399 | MIMAT0000792 | no differentiation | no differentiation |
| **hsa-miR-29a*** | rno-miR-29a-5p | 002447 | MIMAT0004718 | no differentiation | no differentiation |
| **hsa-miR-30a-3p** | rno-miR-30a-3p | 000416 | MIMAT0000809 | no differentiation | no differentiation |
| **hsa-miR-328** | rno-miR-328a-3p | 000543 | MIMAT0000564 | reference | reference |
| **rno-miR-7** | - | 000582 | MI0029387 | no differentiation | no differentiation |
| **mmu-miR-7a-2*** | rno-miR-7a-2-3p | 462860_mat | MIMAT0017091 | no differentiation | no differentiation |
| **dme-miR-7** | rno-miR-7a-5p | 000268 | MIMAT0000606 | no differentiation | no differentiation |
| **mmu-miR-34b-3p** | rno-miR-34b-3p | 002618 | MIMAT0017105 | no differentiation | no differentiation |
| **rno-miR-16*** | rno-miR-16-3p | 462804_mat | MIMAT0017094 | beyond the detection limit | beyond the detection limit |
| **mmu-miR-362-3p** | rno-miR-362-3p | 002616 | MIMAT0017357 | beyond the detection limit | beyond the detection limit |
| **mmu-miR-429** | rno-miR-429 | 001077 | MIMAT0001538 | beyond the detection limit | beyond the detection limit |
| **mmu-miR-451** | rno-miR-451-5p | 001141 | MIMAT0001633 | no differentiation | no differentiation |
| **mmu-miR-674*** | rno-miR-674-3p | 001956 | MIMAT0005330 | no differentiation | no differentiation |
| **hsa-miR-16** | rno-miR-16-5p | 000391 | MIMAT0000785 | no differentiation | no differentiation |
| **rno-miR-1** | rno-miR-1-3p | 002064 | MIMAT0003125 | beyond the detection limit | no differentiation |
| **rno-miR-7*** | rno-miR-7* | 001338 | MI0000263 | no differentiation | no differentiation |
| **rno-miR-7a*** | rno-miR-7a-1-3p | 002062 | MIMAT0000607 | reference | reference |
