# Supplementary material for: Habenula as a Possible Target for Treatment-Resistant Depression Phenotype in Wistar Kyoto Rats
Source: Mol Neurobiol. 2022 Nov 8;60(2):643–54. doi: 10.1007/s12035-022-03103-y (PMC9849162; doi:10.1007/s12035-022-03103-y)
Supplement: Supplementary file 2 — Supplementary file2 (DOCX 21 KB) [file 12035_2022_3103_MOESM2_ESM.docx]

Table 2. The table contains information about investigated mRNA and comparisons expression levels in habenular nuclei in two rat strains - WIS (control group) and WKY (experimental group).

| **Gene Symbol** | **Gene Name** | **Assay ID** | **Expression level MHb** | **Expression level LHb** |
| --- | --- | --- | --- | --- |
| **Cdkn1c** | cyclin-dependent kinase inhibitor 1C | Rn01502044_g1 | significant statistical difference | no differentiation |
| **Drd2** | dopamine receptor D2 | Rn00561126_m1 | no differentiation | significant statistical difference |
| **Elk4** | ETS-domain protein (SRF accessory protein 1) | Rn01484014_m1 | no differentiation | significant statistical difference |
| **Htr2a** | 5-hydroxytryptamine (serotonin) receptor 2A, G protein-coupled | Rn01468302_m1 | beyond the detection limit | significant statistical difference |
| **Htr4** | 5-hydroxytryptamine (serotonin) receptor 4, G protein-coupled | Rn00563402_m1 | no differentiation | significant statistical difference |
| **Htr7** | 5-hydroxytryptamine (serotonin) receptor 7, adenylate cyclase-coupled | Rn00576048_m1 | significant statistical difference | significant statistical difference |
| **Kcnj5** | potassium channel, inwardly rectifying subfamily J, member 5 | Rn01789221_mH | no differentiation | significant statistical difference |
| **Kcnj9** | potassium channel, inwardly rectifying subfamily J, member 9 | Rn00587665_m1 | significant statistical difference | no differentiation |
| **Slc12a5** | solute carrier family 12 (potassium-chloride transporter), member 5 | Rn00592624_m1 | significant statistical difference | significant statistical difference |
| **Sstr4** | somatostatin receptor 4 | Rn00564741_s1 | no differentiation | significant statistical difference |
| **Slc6a6** | solute carrier family 6 (neurotransmitter transporter), member 6 | Rn00567962_m1 | no differentiation | no differentiation |
| **Slc6a4** | solute carrier family 6 (neurotransmitter transporter), member 4 | Rn00564737_m1 | no expression | no expression |
| **Slc6a2** | solute carrier family 6 (neurotransmitter transporter), member 2 | Rn00580207_m1 | no expression | no expression |
| **Slc17a6** | solute carrier family 17 (vesicular glutamate transporter), member 6 | Rn00584780_m1 | no differentiation | no differentiation |
| **Nrg1** | neuregulin 1 | Rn01482165_m1 | no differentiation | no differentiation |
| **Prl8a5** | prolactin family 8, subfamily a, member 5 | Rn01789326_m1 | no expression | no expression |
| **Prl3a1** | Prolactin family 3, subfamily a, member 1 | Rn01434782_m1 | no expression | no expression |
| **Mapk14** | mitogen activated protein kinase 14 | Rn00578842_m1 | no differentiation | no differentiation |
| **Mapk8** | mitogen-activated protein kinase 8 | Rn01218952_m1 | no differentiation | no differentiation |
| **Tph1** | tryptophan hydroxylase 1 | Rn01476867_m1 | beyond the detection limit | beyond the detection limit |
| **Gpr55** | G protein-coupled receptor 55 | Rn03037213_s1 | no expression | no expression |
| **Sstr3** | somatostatin receptor 3 | Rn02134439_s1 | no expression | no differentiation |
| **Sstr2** | somatostatin receptor 2 | Rn01464950_g1 | no differentiation | beyond the detection limit |
| **Gabra1** | gamma-aminobutyric acid (GABA) A receptor, alpha 1 | Rn00788315_m1 | no differentiation | no differentiation |
| **Gabrb1** | gamma-aminobutyric acid (GABA) A receptor, beta 1 | Rn00564146_m1 | no differentiation | no differentiation |
| **Chrna1** | cholinergic receptor, nicotinic, alpha 1 (muscle) | Rn01278033_m1 | no expression | no expression |
| **Ntrk3** | neurotrophic tyrosine kinase, receptor, type 3 | Rn00570389_m1 | no differentiation | no differentiation |
| **Ntrk2** | neurotrophic tyrosine kinase, receptor, type 2 | Rn01441749_m1 | no differentiation | no differentiation |
| **Cacna1b** | calcium channel, voltage-dependent, N type, alpha 1B subunit | Rn00595911_m1 | no differentiation | no differentiation |
| **18S** | Eukaryotic 18S rRNA | Hs99999901_s1 | reference | reference |
| **Ppia** | peptidylprolyl isomerase A (cyclophilin A) | Rn00690933_m1 | reference | reference |
| **Rpl32** | ribosomal protein L32 | Rn00820748_g1 | reference | reference |
